# Sexual Conflict over the Maintenance of Sex: Effects of Sexually Antagonistic Coevolution for Reproductive Isolation of Parthenogenesis

**DOI:** 10.1371/journal.pone.0058141

**Published:** 2013-02-28

**Authors:** Kazutaka Kawatsu

**Affiliations:** Laboratory of Insect Ecology, Graduate School of Agriculture, Kyoto University, Kitashirakawaoiwake-cho, Sakyo-ku, Kyoto, Japan; University of California, Berkeley, United States of America

## Abstract

Sexual reproduction involves many costs. Therefore, females acquiring a capacity for parthenogenetic (or asexual) reproduction will gain a reproductive advantage over obligately sexual females. In contrast, for males, any trait coercing parthenogens into sexual reproduction (male coercion) increases their fitness and should be under positive selection because parthenogenesis deprives them of their genetic contribution to future generations. Surprisingly, although such sexual conflict is a possible outcome whenever reproductive isolation is incomplete between parthenogens and the sexual ancestors, it has not been given much attention in the studies of the maintenance of sex. Using two mathematical models, I show here that the evolution of male coercion substantially favours the maintenance of sex even though a female barrier against the coercion can evolve. First, the model based on adaptive-dynamics theory demonstrates that the resultant antagonistic coevolution between male coercion and a female barrier fundamentally ends in either the prevalence of sex or the co-occurrence of two reproductive modes. This is because the coevolution between the two traits additionally involves sex-ratio selection, that is, an increase in parthenogenetic reproduction leads to a female-biased population sex ratio, which will enhance reproductive success of more coercive males and directly promotes the evolution of the coercion among males. Therefore, as shown by the individual-based model, the establishment of obligate parthenogenesis in the population requires the simultaneous evolution of strong reproductive isolation between males and parthenogens. These findings should shed light on the interspecific diversity of reproductive modes as well as help to explain the prevalence of sexual reproduction.

## Introduction

Sex is an inefficient way to reproduce as it involves many costs [1]–[3] and should thus be vulnerable to invasions by parthenogenetic reproduction. Furthermore, the cost of sex becomes more problematic under the assumption that paternal investment in offspring is minimal, because sexual females are burdened with male production that are completely wasteful in terms of population growth (the cost of males [4]). The fact, however, is paradoxical: obligate sex prevails among anisogamous animals [5], [6], in which males produce smaller gametes by definition. Most studies have focused on genetic mixing or recombination that is specific to sexual reproduction, and such theories showed that recombination does indeed confer some benefits on sexual species [7]–[9]. Also from the entropy point of view, both sexual and asexual reproduction can be regarded as a means to consume free energy in least time, and the least-time free energy consumption in respective environments may determine reproductive modes [10]. However, recent advances revealed that the benefits are insufficient to prevent the invasions of asexuality [11], [12], and the maintenance of sexual reproduction under the cost-of-males condition remains an enigma in evolutionary biology [1], [2], [13].

To solve the paradox of sexual reproduction, it is informative to consider how loss of sex can be achieved [14]. A transition from sexuality to parthenogenesis has been hypothesised to take several evolutionary routes [14]–[16]. One such route is a spontaneous origin of parthenogenesis, which occurs through stepwise mutations in the genes involved in parthenogenesis and reproductive isolation from males [14], [15]. However, although some empirical studies have shown that a capacity for parthenogenetic reproduction can gradually be enhanced through positive selection [17]–[20], spontaneous origins are sparsely distributed amongst animals: most of the existing parthenogenetic organisms are known to be derived from hybridisation between closely related species (a hybrid origin), in which reproductive isolation may be accomplished more easily than the spontaneous origins [14]. Thus, the strength of the reproductive barriers of invasive parthenogens would play a significant role in their evolutionary outcome, and it is important to consider which selection pressures act on individuals in the face of parthenogenesis with incomplete reproductive isolation.

In addition to the above issues related to parthenogenesis, the cost-of-males assumption exerts a different selection pressure on individual fitness. The causal condition of minimal paternal effort eventually strengthens competition for mates among males and would lead to a circumstance in which the two sexes disagree over their common interests [21], [22]. This confrontational situation is termed sexual conflict, and in animals two sexes are known to be in conflict over various reproductive interests [22]. Also the evolution of parthenogenesis may be no exception in that it incurs sexual conflict, because parthenogenetic reproduction enhances female's reproductive outputs but completely deprives of male's genetic contribution to future generations. Therefore, for males, any trait to coercively fertilise parthenogens should increase their fitness, whereas it is beneficial for females to reproduce parthenogenetically. This parasitic view of sex, in which males behave as parasites imposing sexual reproduction on females, has been considered within the context of the ‘evolution’ of sex [23], [24], but many studies have disagreed the role of sexual conflict in the maintenance of sex [25]–[27] because they tacitly assume immediate reproductive isolation between males and parthenogens [6], that is, females have genetically full control over their reproductive modes (sexual or parthenogenetic reproduction). However, this assumption is not always justified for spontaneous origins of parthenogenesis, and there should also be room for male counter adaptation against the reproductive barrier in some modes of parthenogenesis as the following reasons.

Parthenogenesis is coarsely defined as the development of an egg without fertilisation by the male genome, and it encompasses a wide variety of developmental mechanisms [13]. In facultative parthenogenesis particularly, reproductive isolation of parthenogenesis is incomplete: sperm can inseminate eggs that have a capacity for parthenogenetic development. For example, in some facultatively parthenogenetic insects, parthenogenesis rarely occurs in the wild when females mate with males [28], [29]. Additionally, in other modes of parthenogenesis, males could evolve to fertilise parthenogens. Sperm-dependent parthenogenesis is one form of parthenogenesis in which sperm from related species are necessary for egg development. The sperm are usually excluded from the gametes and do not genetically contribute to offspring [14], but fertilisation by sperm sometimes occurs and hence the paternal genome can transfer to the next generation [30]–[32]. That is, the evolution of male coercion to fertilise parthenogenetic females is possible; however, it remains unclear whether the male coercion indeed has a role in maintenance of sexual reproduction in these species.

Even if male coercion to fertilise parthenogens can evolve, it should not simply result in maintenance of sexual reproduction. Studies of sexual conflict have predicted that females should evolve resistance to harmful males to reduce their costs, and the arms race between male and female traits would eventually escalate [33], [34]. Therefore, given that male coercion and a female barrier, both of which affect the reproductive isolation of parthenogenesis, can evolve, antagonistic coevolution between the two sexes is similarly generated, which will strongly affect the evolutionary outcome of the maintenance of sex. In addition, sexual conflict over parthenogenesis between the two traits would involve an additional selection pressure or sex-ratio selection: because the evolution of the reproductive barrier of parthenogenesis leads to an increase in females produced by parthenogenesis, males with more coercive trait that overcomes the mutant barrier will gain higher reproductive success in the population with a female-biased sex ratio. That is, feedback mechanism between sex ratio and parthenogenesis regulates the evolution of female barrier and favours the evolution of male coercion. It has been previously known that female-biases in primary sex ratio increase the growth rate of obligate sexuals and reduce the cost of males in sexual reproduction [4]. In addition, a few studies argued that it is advantageous for mothers to produce more males under parthenogenesis with incomplete reproductive isolation [20], [35]. For example, in species with haplo-diploid sex determination, infection by cytoplasmically inherited bacteria sometimes induces parthenogenetic reproduction, and the resultant female-biased sex ratios may select for mating reluctance in females because it results in production of sons [36]. However, these studies seem to be lacking in terms of counter-adaptation in males against parthenogens, and thus little is known about the combined effect of the antagonistic coevolution for reproductive isolation of parthenogenesis and the resultant sex-ratio selection for males on the persistence of sexual reproduction.

In this study, I investigate sexual conflict over the maintenance of sex described above using the following two mathematical models. First, to examine the effects of antagonistic coevolution between the two traits and the sex-ratio selection on the evolutionary outcome of reproductive modes, I construct an analytical model based on adaptive-dynamics theory that can concurrently address the trait evolution and population dynamics [37]–[39]. The model simply considers a situation in which all females can reproduce parthenogenetically, but their reproductive isolation is incomplete (i.e., facultative parthenogenesis). Then, to relax this assumption and examine a broader array of cases of the maintenance of sex, I further develop an individual-based model that corresponds to the analytical model except for description of evolution of the capacity for parthenogenesis. The results of the models will help to explain the interspecific diversity of reproductive modes as well as demonstrate the significance of the evolution of male coercion for the maintenance of sex.

## Results

### Adaptive dynamics

The model considers a situation of facultative parthenogenesis in which all females have the capacity to reproduce parthenogenetically, but males are able to fertilise females to some extent because female reproductive isolation is incomplete. This is because of the convenience of analysis. In addition, the assumption of facultative parthenogenesis should be justified because I consider no cost of parthenogenetic capacity in this analysis; females with parthenogenetic capacity will gain higher reproductive outputs than obligately sexual females even under the incomplete isolation, and the allele of parthenogenetic capacity should be prevalent in the population. These assumptions are later relaxed in an individual-based model below. The reproductive isolation is assumed to be mediated by two independent sex-limited traits: a female barrier *x* and male coercion *y*. In the model, because frequency of parthenogenetic reproduction in the population depends on the level of reproductive isolation, the values of male coercion and female barrier are associated with the densities of males and female or population sex ratio, and will then affect selection pressure for the two traits in the next generation. To describe this feedback between trait evolution and the population density, the model is developed using adaptive-dynamics theory, which can concurrently analyse population dynamics and evolutionary dynamics [37]–[39].

In the model, individuals are assumed to undergo mating, reproduction, and viability selection within a generation. Mating occurs by the following procedure. A female with trait *x* is fertilised upon encountering a male with trait *y* with a probability determined by the function *ψ*(*x*, *y*)  =  exp[−*α*(*x* – *y*)^2^] (note that *ψ* is a probability for a female to be fertilised per one mating attempt by a male, not probability densities of the distribution of *x* and *y*). This type of function is often used in the models of sexual conflict over reproductive barrier [34], [40], and it indicates that the probability of fertilisation at one mating event is maximised when values of the two traits match and otherwise decreases. The difference in the two traits indicates the degree of reproductive isolation between the two individuals, and the parameter *α* scales the strength of reproductive isolation on the trait difference *x* - *y*. In addition, the net probability of female fertilisation in real organisms would depend on rate or the number of mating interactions which females experience as well as the degree of reproductive isolation from a given male. For example, under mating systems with a large number of mating interactions, it is difficult to avoid fertilisation for a female even though she has a strong barrier against male mating attempt. On the other hand, a female with a weak barrier may easily avoid fertilisation when she undergoes mating interactions less frequently. I assume that the rate of the mating event is simply determined by the potential reproductive rate (PRR) of males and females. The PRR is the rate at which the two sexes are ready to reproduce [41], and hence it should affect the number of mating interactions which a female experiences. In the model, I define the parameter *μ* as the ratio of male PRR to female PRR (i.e., male PRR/female PRR), and it affects female fertilisation with the form *ψ* ^1/*μ*^. That is, under conditions that the PRR is higher in males than in females (*μ*>1.0), females are easily fertilised even though they have an effective barrier against male coercion in the population; otherwise, they can avoid mating and reproduce parthenogenetically with ease.

**Figure 1:**
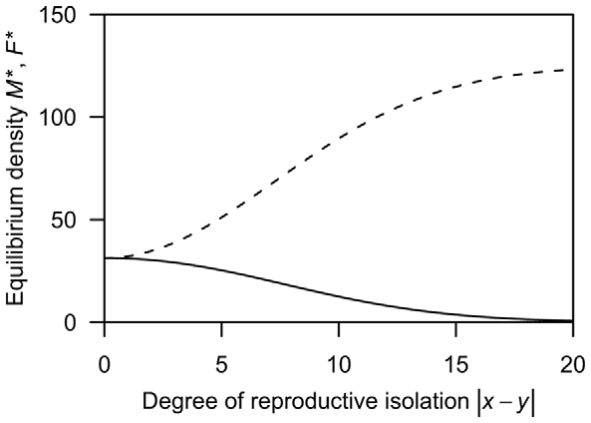
Population densities of the two sexes as a function of the degree of reproductive isolation. Solid and dashed lines indicate the densities of males and females, respectively. Parameters used here are *r*  = 1.25, *h*  = 0.01, *α*  = 0.01, *μ* = 1.0, and *β_m_*  =  *β_f_*  = 0.001.

Fertilised females sexually produce sons and daughters with equal probability, and unfertilised females produce only daughters parthenogenetically. For viability selection, I assume that investing in male coercion or a female barrier imposes a mortality cost on males and females, respectively. The phenotype-dependent mortalities are determined by the following functions: *c_m_*(*y*)  = 1 – exp[−*β_m_y* ^2^] for males and *c_f_*(*x*)  = 1 – exp[−*β_f_x* ^2^] for females (also note that *c_m_*(*y*) and *c_f_*(*x*) are mortalities, not probability densities of the distribution of *x* and *y*). That is, individuals incur harsher mortality costs with increased investment in the trait. The parameter *β_m_* and *β_f_* scales a selection coefficient of mortality for male coercion and a female barrier, respectively. The non-negative equilibrium densities of males and females (*M* ^*^ and *F* ^*^) in this system is obtained as
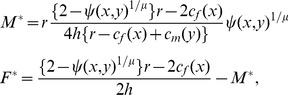
(1)where the parameter *r* and *h* indicates the intrinsic birth rate and the density-dependent mortality, respectively (see Methods). These equations indicate that equilibrium densities of males and females depend on the degree of isolation which is determined by the values of *x* and *y* in the population. Therefore, the population sex ratio becomes *F* ^*^/M^*^  = 1–2(*cf*(*x*) – *cm*(*y*))/*r* if male coercion can evolve to eliminate the reproductive isolation between males and females (i.e., *x*  =  *y*), but otherwise the male population will go extinct (Fig. 1).

Next, I investigate trait evolution under varying population sex ratios. To this end, the model addresses the situation in which rare individuals with a mutant trait invade the equilibrium population (equation 1), in which all resident individuals have an identical value for the two traits [37]. Assuming that the densities of males and females rapidly reach equilibrium under given trait values [37]–[39], the selection gradients for the female barrier and male coercion can be derived as follows (see Methods):
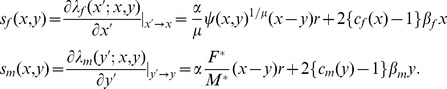
(2)


The equation (2) indicates that the selection gradient for male coercion is also affected by the population sex ratio (i.e., F*/M*), whereas the gradient for the female barrier is determined by only the trait values. As I am interested in coevolution between male coercion and the female barrier, these selection gradients are dependent both on the values of *x* and *y*. Coevolutionary dynamics of male coercion and the female barrier are determined by equation (1) and the equations *dx*/*dt*  =  *δsf*(*x*) and *dy*/*dt*  =  *δsm*(*y*)delta modified the equation as line 9, where the parameter *δ* scales the rate of trait evolution.

**Figure 2:**
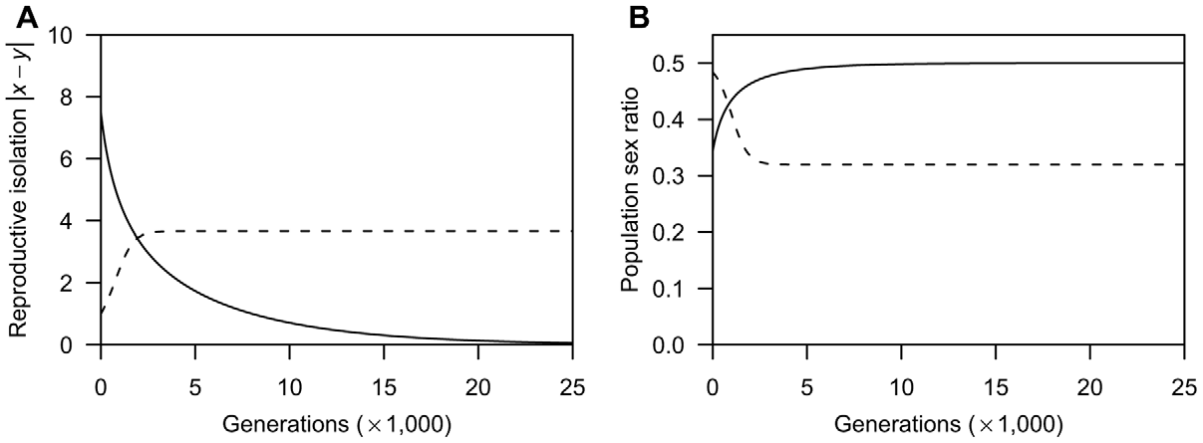
Examples of evolutionary dynamics of male coercion and the female barrier over 25000 generations under the assumption of no mortality costs. **A**: The coevolutionary dynamics of the degree of reproductive isolation; **B**: The demographic dynamics of the population sex ratio. The solid line indicates the dynamics in a population with higher male PRR (*μ* = 1.5) that starts at (*x*, *y*)  =  (7.5, 0.0). The dashed line indicates the dynamics in a population with lower male PRR (*μ*  = 0.3) that starts at (*x*, *y*)  =  (1.0, 0.0). Other parameters are *r* = 2.5, *h* = 0.001, *α* = 0.01, and *δ* = 0.02.

**Figure 3:**
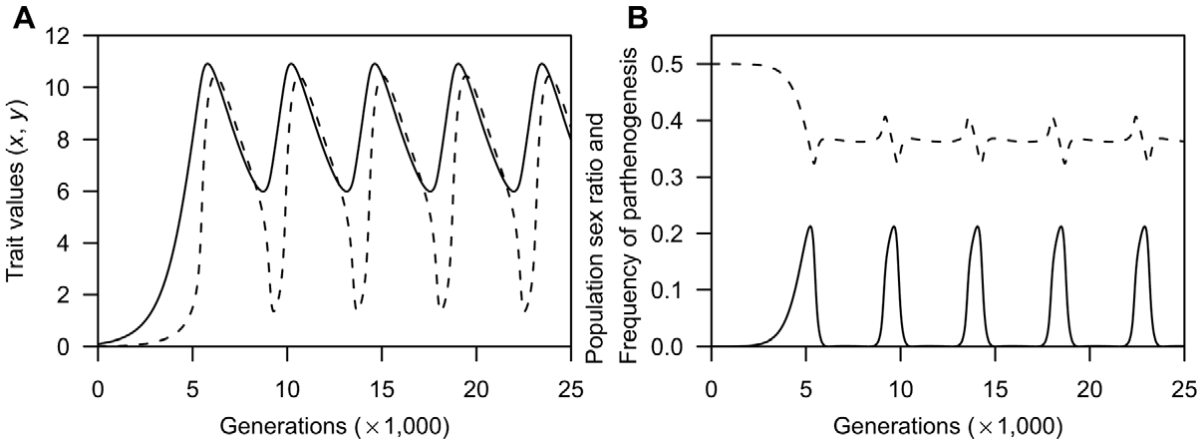
Example of oscillation dynamics over 25,000 generations with the mortality costs of trait investment. **A**: The coevolutionary dynamics of male coercion and the female barrier (indicated by the solid and dashed line, respectively). **B**: The demographic dynamics of the frequency of parthenogenetic reproduction and the population sex ratio (indicated by the solid and dashed line, respectively). Because of positive values of mortality coefficients (*β_m_*  = 0.1, *β_f_*  =  0.001), trait investment inflicts mortality costs on individuals. The population starts at (*x*, *y*)  =  (0.1, 0.0) under the condition of higher male PRR (*μ* = 2.0). Other parameters are as in Figure 2.

**Figure 4:**
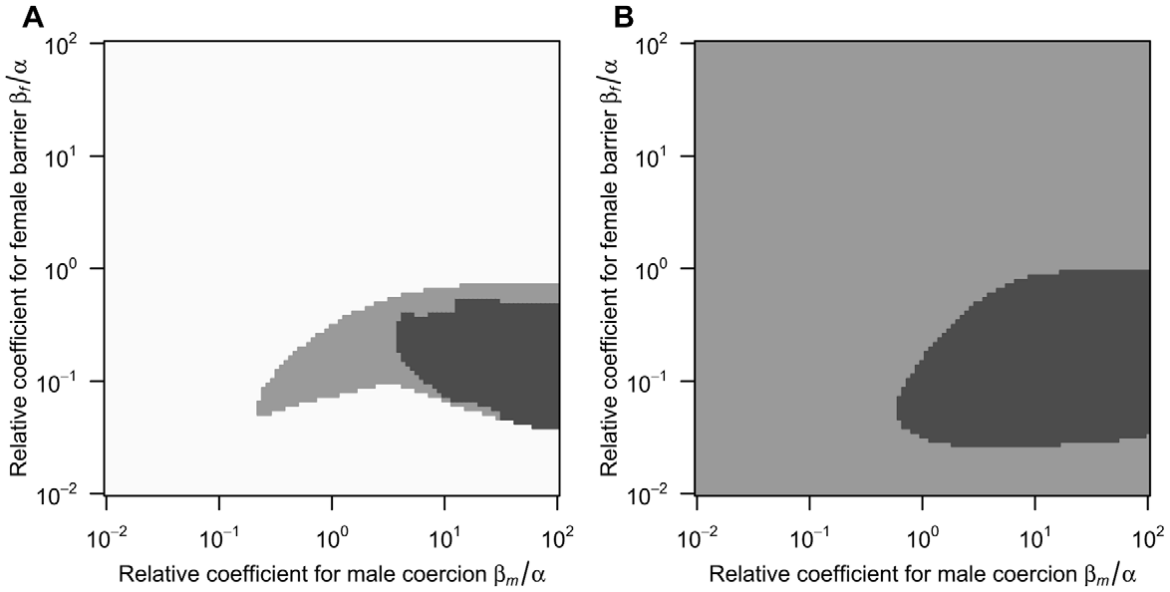
Numerical calculation of the adaptive-dynamics model. Each panel shows evolutionary outcomes of the antagonistic coevolution after 100,000 generations under various ratios of the mortality coefficients to the coefficient of female fertilisation (*β_f_*/*α* and *β_m_*/*α*) The two panels differ in the value of male PRR (**A**: *μ* = 2.00, **B**: *μ* = 0.50). Each coloured region indicates a different evolutionary outcome (white: prevalence of sexual reproduction, light grey: co-occurrence of two reproductive modes; dark grey: oscillation of the occurrence of parthenogenesis). Other parameters are as in Figure 2.

Because it is hard to analytically obtain candidate equilibria of this system using full versions of equation (2), I first analyse the case of no mortality cost to trait investment (i.e., *β_m_*  =  *β_f_*  = 0.0). In this case, solving *s_f_*(*x*)  = 0 and *s_m_*(*y*)  = 0 yields an analytical solution in which values of the two traits are equal (i.e., *x* = *y*). Stability analysis [42] demonstrates that these equilibria are stable whenever the inequality *μ*≥1.0 is satisfied (see Methods for the detail of the analysis). The solid lines in figure 2 represent an example of coevolutionary dynamics of male coercion and the female barrier in this case; even if a mutant female with an enhanced reproductive barrier invades, reproductive isolation from males will be lost under conditions of higher male PRR (Fig. 2A). Thus, sexual reproduction is eventually imposed on all females and the population sex ratio becomes balanced (Fig. 2B). However, when *μ*<1.0, a different system arises. Evolutionary rates of the two traits necessarily become equal at a value of 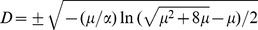, which is obtained by solving *s_f_*(*x*)  =  *s_m_*(*y*), and thus the difference between the two traits approaches either the value *D*, depending on initial trait values (Fig. 2A; the dashed line). At this value, both traits keep changing in the same direction at a constant rate, and females reproduce parthenogenetically with a probability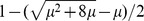: the population sex ratio remains biased toward females (Fig. 2B; the dashed line). That is, in the case of no mortality cost, the coevolution between male coercion and the female barrier shows the following two dynamics depending on the value of male PRR: evolution to the line of equilibria *x* = *y* when *μ*≥1.0 and continuous increase in *x* and *y* at a constant rate when *μ*<1.0. These are similar to the dynamics shown in a previous study that investigated the sexually antagonistic coevolution of reproductive barrier in sexual species [34].The above analysis reveals that the antagonistic coevolution for reproductive isolation results in only two outcomes (i.e., the prevalence of sex and the co-occurrence of two reproductive modes) under the absence of natural selection on trait investment. Next, to investigate how the mortality costs of trait investment affect the frequency of parthenogenetic reproduction, I perform numerical calculations that track population densities and the degree of reproductive isolation based on equations (1) and (2), under various ratios of the mortality coefficients to the coefficient of female fertilisation (*β_m_*/*α* and *β_f_*/*α*). The numerical analysis indicates that additional mortality costs yield a new outcome of parthenogenesis: because both male coercion and the female barrier display periodic dynamics that are out of phase with each other (Fig. 3A), the frequency of parthenogenetic reproduction and the population sex ratio periodically oscillate (Fig. 3B; the solid line and dashed line, respectively). In the case of both lower and higher male PRR, this outcome arises under conditions where the mortality coefficient of the female barrier is moderately lower and that of male coercion is higher than the coefficient of female fertilisation (Fig. 4; indicated by the dark grey region). In addition, the co-occurrence outcome (the light grey region) arises for the first time under higher male PRR around oscillation outcome (see Fig 4A). These results also show that male population persists even under disadvantageous conditions for them, such as lower male PRRs and a larger mortality coefficient for male coercion than that for the female barrier (i.e., *β_f_*/*α* < *β_m_*/*α*). Please note that these oscillation dynamics emerging from interactions of sexual conflict and viability selection are similar to the dynamics in a previous study of sexual conflict in obligate sexuals [43].

### Analysis of individual-based model

The above analysis considers only the case of facultative parthenogenesis, in which the capacity for parthenogenesis is prevalent and fixed in the population, under unspecified genetic mechanisms. In the second analysis, to relax these assumptions and examine antagonistic coevolution in more diverse and more realistic situations of the maintenance of sex, I develop a corresponding individual-based model that incorporates evolution of parthenogenesis and a diploid genetics. To this end, I introduce alleles that determine female reproductive capacity (an obligately sexual allele *a* and a parthenogenetic allele *A*) in addition to the alleles for male coercion and the female barrier (for more detail, see Methods). The model addresses circumstances in which an allele of parthenogenetic capacity and some reproductive barrier simultaneously evolve in one female (the invasive parthenogen; see Methods). The resident population consists of obligately sexual individuals (1000 males and 1000 females), and thus the value of the two traits are optimised for mating and viability (i.e., *x* = *y* = 0.0). In simulation runs, the frequency of parthenogenetic reproduction, the frequency of parthenogenetic alleles, and the degree of reproductive isolation between mean values of the two traits |*x* – *y*| are monitored. If not specified otherwise, the simulation is iterated 25 times for each parameter set.

**Figure 5:**
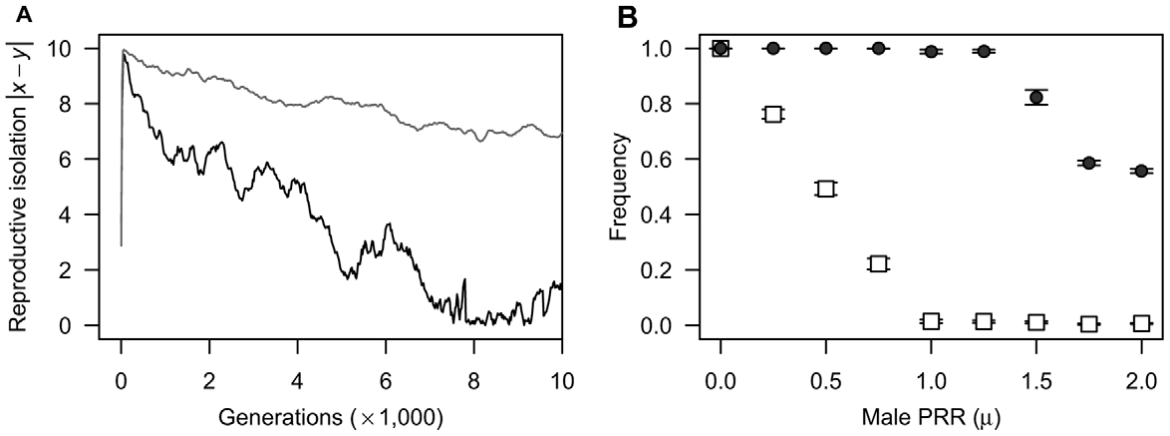
Simulation results of the individual-based model in the case of no cost of parthenogenesis. **A**: Example dynamics of the degree of reproductive isolation for 10,000 generations in a single simulation run. Black and grey lined differ in the value of male PRR (the black line: *μ* = 1.25, the grey line: *μ* = 0.25). **B**: Mean frequency of parthenogenesis after 10,000 generations as a function of male PRR. Open-squares and filled-circles represent the frequency of females succeeding in parthenogenetic reproduction and the frequency of parthenogenetic alleles, respectively. Bars indicate standard deviation over 25 replicates. Other parameters are *r* = 10.0, *h* = 0.001, *α*  = 0.01, *β_m_*  = *β_f_*  = 0.0001.

As shown in examples of evolutionary dynamics of reproductive isolation for a single simulation run (Fig. 5A), the degree of reproductive isolation from males is initially high because of spreads of the invasive parthenogen. However, as generation proceeds, reproductive isolation vanishes from the population under the condition of higher male PRR (the black line), or approaches a fixed value under the condition of lower male PRR (the grey line). Therefore, the frequency of females that succeed in parthenogenetic reproduction decreases as s function of male PRR, and sexual reproduction is imposed on most females when *μ*≥1.0 (Fig. 5B; indicated by the open-squares). Moreover, I confirm that the IBM with the condition of facultative parthenogenesis yields results quantitatively similar to those of the adaptive-dynamics model under same parameter set (see Fig. S1). Therefore, the antagonistic coevolution for reproductive isolation works to maintain the male population under the assumption of the individual-based model, as in the adaptive-dynamics model.

**Figure 6:**
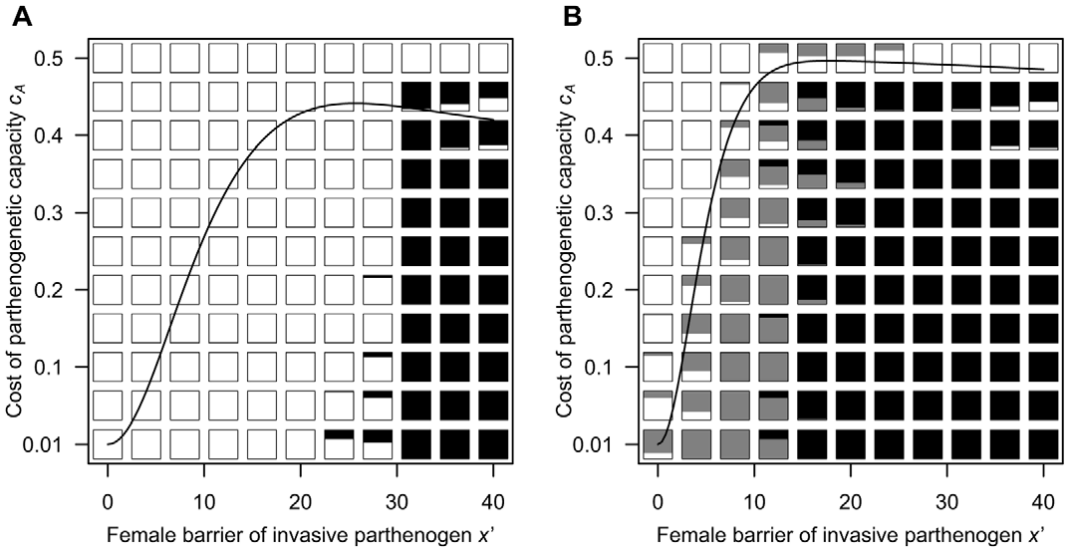
Simulation outcomes after 20,000 generations under various values of the reproductive barrier of the invasive parthenogens and the cost of parthenogenetic capacity. The two panels differ in the value of male PRR (**A**: *μ* = 2.00, **B**: *μ* = 0.50). Each box indicates the proportion of different outcomes over 25 replicates under its parameter set (white: the obligate-sex outcome; grey: the facultative-parthenogenesis outcome; black: the obligate-parthenogenesis outcome). The solid curves are the analytical threshold of *c_A_* for successful invasion by the invasive parthenogen (see Methods). Other parameters are as in Figure 5.

Figure 5B also demonstrates that the frequency of parthenogenetic alleles (the filled-circles) moderately decreases when *μ*>1.5. This is because the possibility that females succeed in parthenogenesis is strongly reduced, and selection for the parthenogenetic capacity becomes neutral. Thus, parthenogenetic alleles are predicted to be purged if they are deleterious for individual fitness. To verify this prediction, I incorporate a fitness cost *c_A_* into an allele for parthenogenetic capacity in the individual-based model (see Methods), and perform additional simulations under various costs of parthenogenesis *c_A_* and values of female barrier of the invasive parthenogen *x*'. The simulation run ends in one of the following three outcomes: in the first, parthenogenetic alleles go extinct and most of the population consists of only obligate sexuals (the obligate-sex outcome); in the second, the parthenogenetic allele becomes prevalent in the population but sexual reproduction is imposed on some proportion of females by coercive males (the facultative-parthenogenesis outcome); in the third, males are extinct and females reproduce only parthenogenetically (the obligate-parthenogenesis outcome). Specifically, under the condition of higher male PRR (Fig. 6A), the obligate-sex outcome dominates even with a small cost of parthenogenetic capacity, and occurs in broader regions than those which are expected by the analytical threshold (the solid curve; see Methods). When male PRR is lower than female PRR (Fig. 6B), the obligate-sex outcome occurs in limited regions with a large cost of parthenogenesis and/or a small value of female barrier of invasive parthenogen, which are qualitatively consistent to those of the analytical threshold. The simulation also demonstrates that the occurrence of the obligate-parthenogenesis outcome requires significantly larger reproductive barriers than those of the threshold for both conditions of male PRR (Fig. 6).

## Discussion

In summary, the theoretical framework suggests that the evolution of male coercion substantially promotes conditions for the maintenance of sex even though it exerts direct selection for a female barrier. The analysis of adaptive-dynamics model demonstrates that the resultant antagonistic coevolution for reproductive isolation fundamentally results in one of the two outcomes depending on the value of male PRR, and males persist under both outcomes. When males are ready to reproduce faster than females (higher male PRR) and thus many mating interactions occur in the population, selection for male coercion is stronger than that for the female barrier and the male trait can overcome the female barrier with ease. Furthermore, even under conditions of lower male PRR, both modes of reproduction co-occur in the population because selection pressure on the two traits will necessarily be equal at a certain level of reproductive isolation.

This interesting result stems from the fact that the coevolution between the two traits is additionally affected by sex-ratio selection (see equation 2), which counterbalances the difference in selection pressure: sex-ratio selection regulates the evolution of female barrier and selects for male coercion because an increase in the barrier leads to female-biased sex ratios in the population and thus more coercive males gains higher reproductive success. The impact of sex-ratio selection is well illustrated in the examples of oscillation dynamics (Fig. 3). The oscillation dynamics tends to occur under conditions of mortality coefficient for male coercion higher than that for the female barrier (Fig. 4; the dark grey regions). Therefore, male coercion would evolve slower or be lost faster than would the female barrier (Fig. 3A), and females succeed in parthenogenetic reproduction with ease. Furthermore, because an increase in the trait investment inflicts a harsher cost on males than on females, the male density decreases faster than the female density. These processes easily bias the population sex ratio toward females (Fig, 3B), and eventually generate sex-ratio selection that promotes the evolution of male coercion. That is, if males are capable of evolving to fertilise parthenogens in some way, the maintenance of sex would be robust due to the sex-ratio selection even under disadvantageous conditions for the evolution of the male trait (lower male PRR and/or higher investment cost to males).

Based on these conclusions, the establishment of obligate parthenogenesis will require that females simultaneously evolve both the capacity for parthenogenesis and complete reproductive isolation from males of the parent sexual species. The results of the individual-based simulation confirm this prediction: the invasive parthenogen drives out the sexual population only when she initially has a strong reproductive-barrier (Fig. 6). This prediction is important because it may explain why many parthenogenetic lineages in animals have a hybrid origin [14]. As described above, although the spontaneous origin is hypothesised as one possible route to the loss of sex [14], [15], this would be a rare event compared to other origins of parthenogenesis [14]. In many taxa, a small fraction of unfertilised eggs occasionally parthenogenetically develop into viable zygotes [13], [14]. In addition, some studies of insects have shown that the capacity for parthenogenesis can gradually be enhanced through positive selection for unfertilised females that reproduce parthenogenetically [17], [18], [20]. However, these parthenogenetic females easily return to sexual reproduction when they mate with males, and an obligately parthenogenetic population has not been found in these species in the wild [19], [20]. These observations may result from incomplete isolation in parthenogenetic reproduction: sperm can easily fertilise eggs produced by parthenogens, because in most of these species, haploid eggs are necessarily produced during parthenogenesis (automixis) [14], [44], and selection pressure may only be exerted on the ability for parthenogenesis but not on reproductive isolation. In this case, parthenogenesis cannot confer the reproductive advantage on females and both sexes may share mutual interest over sexual reproduction owing to rarity of parthenogenetic development or less fecundity in parthenogenesis. On the other hand, a hybrid origin, which is the other route to parthenogenesis [14], [16], may provide simultaneous reproductive isolation. Owing to the disruption of meiosis, daughters of interspecific hybridisation frequently produce unfertilised diploid eggs, and parthenogenetic species are readily established if these eggs succeed in normal embryogenesis [45]. In this situation, males will be extinct even if they can fertilise parthenogenetic females, because fertilisation by sperm frequently results in the evolution of a new parthenogenetic species with polyploidy [14].

The models presented here further demonstrate that sexual reproduction is prevalent in mating systems with higher male PRR (Fig. 2, 4, and 5), and the capacity for parthenogenesis is lost if it inflicts any cost on females (Fig. 6A). These results should offer a possible explanation for the paradoxical prevalence of obligate sex among organisms with anisogamy or the cost-of-males assumption. In general, male PRR would increase under conditions of anisogamy [41], and obligate sexual reproduction is actually common in animals [13], [45], which are characterised by a high degree of anisogamy [21], [22]. In contrast, under conditions of isogamy (equal gamete sizes and comparable PRRs between sexes), parthenogenesis may evolve frequently (Fig. 2, 4, 5, and 6B); some reviews point out that despite the lower cost of producing males, parthenogenesis is more frequent in isogamous organisms than in anisogamous ones [5], [6].

Although these predictions may explain important issues related to the maintenance of sex, they require that the evolution of male coercion can occur in real parthenogenetic systems. As described above, the reproductive barrier of parthenogenetic females may be incomplete – this is more likely for facultative and/or automictic parthenogenesis than for other modes of parthenogenesis, and may evolve to penetrate the incomplete barrier. Automixis and facultative parthenogenesis occur in many insects, and females of these species reproduce both sexually and parthenogenetically under experimental conditions [20], [28], [29]. However, although female fecundity or offspring viability under parthenogenesis are not less than half of those under sexual reproduction in some species with facultative parthenogenesis, it is reported that most females are mated in the wild and rarely reproduce parthenogenetically [29], [46], [47]. For example, in the Japanese common termite *Reticulitermes speratus*, females or queens have the ability for automixis and can parthenogenetically produce offspring in numbers comparable to those produced sexually [46]: if the queen could sneakily perform parthenogenetic reproduction, she should enhance her reproductive output. However, parthenogenesis rarely occurs within wild colonies (but see [48]), implying that any coercion or counter-adaptation by males may inhibit sneaky females in this species.

The evolution of male coercion might also occur in other forms of parthenogenesis, such as gynogenesis or hybridogenesis. These parthenogenetic modes are known as sperm-dependent parthenogenesis and are often found in teleost fishes [31], [32]. The sperm-dependent species consist of only females, and sperm from related bisexuals are required for egg development but do not genetically contribute to gamete production [14]. However, if the male genomes can evolve to transmit to the next gametes, this mutation will confer a reproductive benefit to the males. Interestingly, genetic contributions of the paternal genome have been observed in many species to various degrees, from partial to entire chromosome sets (paternal leakage) [32]. In extreme cases of some stick insects, maternal genes are excluded from the gametes and only the paternal genomes are passed on to the offspring [28], [49]. In these reproductive modes, females of sperm-dependent species produce both fertile male and female offspring, and they may revert to sexual reproduction by mating with the males [28], [32], [50]–[52].

In addition, similar selection pressure might occur in reproductive modes other than parthenogenesis. In androdioecy, a population consists of males and hermaphrodites. A study of androdioecious shrimp demonstrated that hermaphrodites are capable of selfing and outcrossing with males but cannot fertilise other hermaphrodites [53]. Thus, this system is analogous to automixis [54]: in outcrossing, hermaphrodites act as females and incur the cost of males. A model based on this system shows that the maintenance of males requires a substantial cost of selfing and a high fecundity of males [54] but an empirical study reported that estimates of selfing costs do not satisfy the model's prediction of maintaining androdioecy [55]. Interestingly, some mating efforts of males to increase fertilisation success have reported in this androdioecious shrimp [56]. For example, males attempt to overcome pre-mating barrier of hermaphrodites by thrusting his tail into hermaphrodite's body [56]; mate guarding should also have a role in the improvement of reproductive success of males in post-mating period [57]. I suggest that these mating behaviour of males and the resultant sexually antagonistic coevolution for selfing/outcrossing similarly promotes the persistence of males in this system.

The important factor yielding the result of this study, i.e., the sexually antagonistic coevolution over reproductive isolation of parthenogenesis is fundamentally ends in maintaining sexuality, is the sex-ratio selection for male coercion. Therefore, although only the particular form is used as the function of female fertilisation in the models, the main result should hold regardless of forms of reproductive isolation if male coercion against the female barrier is able to evolve in some way. Note that, however, there would be some modes of parthenogenesis in which the male counter-adaptation is difficult to evolve. For example, in oribatid mites that are known for the frequent occurrence of obligately parthenogenetic species, female fertilisation is usually indirect and physically isolated from males because it occurs via spermatophores deposited by males in the absence of females [58]. In addition, I assume that the encounter rate is simply determined by the relative male PRR in the models; however, it may depend on the frequency or density of males, and in such cases, parthenogenetic females should be more frequent in low-density and/or female-biased populations even with male coercion (see Fig. S2). A study of stick insects with facultative parthenogenesis demonstrated a negative correlation between population density and the proportion of parthenogenesis, perhaps because positive feedback of parthenogenesis exists between mate limitation and the female-biased sex ratio [20]. The significance of these factors for the evolution of parthenogenesis is unclear, and further studies will contribute to comprehension of the diverse reproductive modes.

Most studies of the maintenance of sex have hypothesised that genetic mixing, which may be a by-product of sexual reproduction, is an advantage of sex [7]–[9]. Although they do not necessarily contradict my models, the explanatory power of recombination as a short-term advantage for sex may attenuate. As illustrated above, in mating systems with higher male PRR, sexual reproduction is imposed on parthenogenetic females, who then enjoy the benefit of recombination. In this situation, it will be important for the prevalence of obligate sex to consider the cost of parthenogenetic ‘allele’ itself [15], [59], as shown by the individual-based model (Fig. 6A). Some authors have reported a reduction in successful offspring [20], [60], [61] when parthenogenetic females produce sexually. These costs stem from the complex mechanisms of sexual reproduction [59], [61], [62], and it should be difficult for mutant parthenogens to arise from sexual populations without these costs. Therefore, in conclusion, organisms with complex sexuality may actually be locked into sexual reproduction by the nature of males rather than because a cost-benefit balance maintains sexual reproduction.

## Methods

### Population dynamics of males and females

I assume that the densities of males and females depend on trait values of reproductive isolation, as they affect mating probability and individual mortality, and are additionally regulated by the crowding effect or the density-dependent mortality exerted by others. In the model, females are assumed to produce *r* offspring per unit time. Denoting the density of males and females by the symbols *M* and *F*, respectively, the equations for describing the population dynamics of males and females become



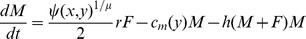
(3a)

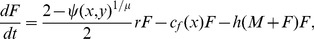
(3b)where the parameter *h* represents the crowding effect [63]. Solving *dM*/*dt*  = 0 and *dF*/*dt*  = 0, equation (3) has at most three potential equilibrium conditions depending on the parameters: (*M* ^*^, *F* ^*^)  =  (0, 0), (−*c_m_*(*y*)/*h*, 0), and the equation (1). Because the population densities are not positive value in the first two conditions, only equation (1) is a meaningful equilibrium for the invasion analysis of the reproductive isolation of parthenogenesis. Stability analysis of these equilibrium are analysed by calculating the Jacobian matrices and determining their eigenvalues. Solving the characteristic polynomial of the Jacobian matrix, dominant eigenvalues for the equilibrium are obtained as (*r*(2 – *ψ*(*x*, *y*)^1/*μ*^) – 2*c_f_*(*x*))/2, (*r*(2 – *ψ*(*x*, *y*)^1/*μ*^) – 2*c_f_*(*x*) + 2*c_m_*(*y*))/2, and –(*r*(2 – *ψ*(*x*, *y*)^1/*μ*^) – 2*c_f_*(*x*))/2, respectively. Therefore when the inequality *r*(2 – *ψ*(*x*, *y*)^1/*μ*^)/2 > *c_f_*(*x*) is satisfied, equation 1 is a single stable equilibrium.

### Evolution of male coercion and a female barrier

The analysis is based on the assumption of the sufficient separation between ecological and evolutionary timescales [37]–[39], [64]. Thus the densities of males and females rapidly reach their equilibrium. Then the obtained equilibrium densities are assigned to the coevolution of male coercion and the female barrier. Consider the situation in which rare mutant individuals consisting on both a male with trait value *y*' and a female with trait value *x*' arise in a population dominated by the resident trait values *x* and *y*. In this case, the invasion fitness of a mutant female with trait value *x*' in the resident population is obtained from equation (3b) as
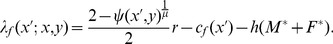
(4)
Equation (4) represents the per capita growth rate of a mutant female with *x*', and it indicates that the invasion fitness increases as her reproductive barrier deviates from resident *y*, but an excessive investment in the barrier reduces her fitness. For the evolution of male coercion, a mutant male gains greater reproductive success as his trait value of coercion approaches that of the resident female barrier. In addition, mating opportunities of males are affected by the strength of competition for mates among males, and male's reproductive success should be evaluated relative to that of other males [64]. Therefore, selection on male coercion *y* is frequency dependent because a male's reproductive success depends not only on his own value of coercion but also on the value of other males, and the invasion fitness of a mutant male with trait value *y*' in the resident population becomes
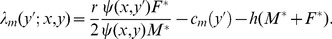
(5)where the first term indicates the reproductive success of the mutant male with the coercion y'. Using equations (4) and (5), the selection gradients (equation 2) are obtained. Equations (2, 4, 5) are based on selection gradients, and hence equivalent results should be obtained if the quantitative genetics method [65], [66] were instead applied to the model, with the assumptions of uncorrelated traits and small additive genetic variances [64]. These genetic assumptions will be relaxed in the following individual-based model (as shown in Fig. S1, the results are quantitatively similar between the two models with same condition).

### Stability analysis of coevolution between male coercion and the female barrier

In the case of no mortality cost to trait investment (i.e., *β_m_*  =  *β_f_*  = 0.0), coevolution between male coercion and the female barrier has lines of equilibria *x*  =  *y*. Stability analysis of these equilibria can be analysed by calculating the Jacobian matrix of equation (2) near the line of equilibria and determining its eigenvalues. The Jacobian matrix becomes.
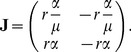
(6)


Solving the characteristic polynomial of the Jacobian matrix (6), a dominant eigenvalue is obtained as *rα*(1–*μ*)/*μ*, and the lines of equilibria is stable when the dominant eigenvalue is smaller than zero. Thus, since the values of *r* and *α* are always positive and the line of equilibria, the condition of stability is obtained as an inequality *μ* ≥1.0. When the inequality is not satisfied (i.e., *μ*<1.0), the line of equilibria *x*  =  *y* is unstable, and the difference between the two traits *D* = *x*–*y* increases. However, evolutionary change in *x* and *y* should be constant when evolutionary rates of two traits become equal. Solving *s_m­_*(*y*)  =  *s_f_*(*x*), candidates of such a trait difference are obtained as 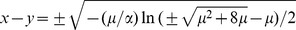. In this analysis, because trait difference should be real number, constant trait difference *D* described above is obtained.

### The individual-based model

In the individual-based model, each individual has three unlinked loci: the first two loci control the continuous traits of reproductive isolation described above, and the third is a newly-introduced locus that codes for a female capacity for parthenogenetic reproduction. The model assumes diploid heritability. For the continuous traits, the alleles have only additive effects and the phenotypes of the female barrier and male coercion are simply determined by the alleles at each locus (i.e., *x* =  (*x*_1_+*x*_2_)/2 and *y*  =  (*y*_1_+*y*_2_)/2, respectively). The capacity for parthenogenesis is determined by two alleles of major effect. The first is an obligately sexual allele *a*, and the other is a parthenogenetic allele *A*. I assume that the parthenogenetic allele *A* has a dominant effect. Thus, a female with at least one parthenogenetic allele has a capacity for parthenogenesis, but otherwise females reproduce only sexually.

In the model, generations are assumed to be discrete and non-overlapping. Three distinct events occur within each generation: mating/reproduction, viability selection, and population-regulation events. During mating, the probability of the fertilisation of a female *ψ* ^1/*μ*^ is determined by her barrier phenotype and mean phenotypic value of coercion among males. For each fertilised female, a male is randomly assigned as a potential father of her offspring, and this operation will be repeated until mating is completed, depending on the probability *ψ* of her barrier and the coercion phenotype of a given male. Therefore, the trait values affect a sex ratio in the population, and then the population sex ratio impacts on male mating success.

Fertilised females reproduce sexually regardless of parthenogenetic capacity genotype, and unfertilised females with the parthenogenetic allele reproduce parthenogenetically. Each female has the potential to produce *r* offspring. However, to take into account costs of parthenogenesis such as delayed hatching or a failure of normal meiosis, which are observed in sexual reproduction by females with parthenogenetic capacity [20], [60], [61], the number of offspring produced by females with the parthenogenetic allele is down-weighted by a factor of 1 – *c_A_* (*c_A_* denotes the fecundity cost of parthenogenetic capacity). In the process of sexual reproduction, offspring equally inherit all alleles from their parents with free recombination, and their sex is randomly assigned (i.e., equal primary sex ratio). In parthenogenetic reproduction, all offspring are female and they clonally inherit alleles from their mothers. Mutations occur at the time of reproduction: the two continuous traits of reproductive isolation (*x*, *y*) are added by mutation based on normal distributions with means of 0.00 and standard deviations of 0.025. Mutation between alleles of parthenogenetic capacity occurs mutually at a rate of 1.0×10^−5^.

After reproduction, selection and population-regulation processes come into operation. Male and female offspring incur the mortality costs determined by their phenotypic values of male harassment and the female barrier, as described above. In the individual-based model, I use the fixed number of population size *N* (if not specified otherwise, *N* = 2000). Thus, denoting the number of surviving offspring at a generation *t* by *N_t_*, surviving offspring are then randomly selected to form the next generation by density-dependent mortality 1– *N*/*N_t_* to maintain the constant population size.

### Threshold of cost of parthenogenesis for successful invasion of the invasive parthenogen

In the initial population, the invasive parthenogen succeed in parthenogenetic reproduction with a probability of 1– *ψ*(*x*', 0)^1/*μ*^ but suffers a mortality cost *c_f_*(*x*') and fecundity cost of parthenogenetic capacity *c_A_*. Therefore, without the evolution of male coercion, the parthenogen can invade the population if her fitness is greater than that of resident females *r*/2– *h*(*M* + *F*). From equation (4), the invasion fitness of the invasive parthenogen becomes *λ_f_*(*x*', *A*; 0, a)  =  *r*(1– *c_A_*){2– *ψ*(*x*', 0)^1/*μ*^}/2– *c_f_*(*x*') – *r*/2, and the threshold of *c_A_* for a successful invasion of the parthenogen is described by the following inequality (the analytical threshold):
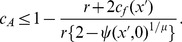
(7)


This inequality indicates that unless the evolution of male coercion is allowed, the invasive parthenogen with a moderate reproductive barrier succeeds in invasion and finally drives out the sexual population (the obligate-parthenogenesis outcome) even if the cost of parthenogenesis is relatively high, but this possibility slightly decreases with an increase in trait investment (Fig. 6).

## Supporting Information

Figure S1 **Simulation results of the individual-based model under the condition of facultative parthenogenesis.** **A**: The coevolutionary dynamics of the mean degree of reproductive isolation over 25 simulation runs; **B**: The demographic dynamics of the mean population sex ratio over 25 simulation runs. The black line indicates the dynamics in a population with higher male PRR (*μ* = 1.5) that starts at (*x*, *y*)  =  (7.5, 0.0). The grey line indicates the dynamics in a population with lower male PRR (*μ* = 0.3) that starts at (*x*, *y*)  =  (1.0, 0.0). Other parameters are *r*  = 10.0, *h* = 0.001, *α*  = 0.01, *β_m_*  =  *β_f_*  = 0.00. In this analysis, the individual-based model assumes the situation of facultative parthenogenesis: all individuals have only parthenogenetic capacity and the mutation does not occur.
(TIF)

Figure S2 **Simulation outcomes of the models with the frequency-dependence under various values of the reproductive barrier of the invasive parthenogens and the cost of parthenogenetic capacity.** In this analysis, I have newly incorporated the frequency-dependence on the fertilization probability of females: male mating attempts are dependent on the population sex ratio and the frequency-dependence affects female fertilisation with the form *ψ*(*x*, *y*)*^M^* ^*/μF*^. As shown here, the frequency-dependence reduces areas of the obligate-sex outcome in comparison with the results of the non-frequency-dependent model (Fig. 6). However, the conclusion of the new model is not quantitatively different with that of the original model: the establishment of obligate parthenogenesis requires that females simultaneously evolve both the capacity for parthenogenesis and complete reproductive isolation from males of parental sexuals. The two panels differ in the value of male PRR (**A**: *μ* = 2.00, **B**: *μ* = 0.50). Each box indicates the proportion of different outcomes over 25 replicates under its parameter set (white: the obligate-sex outcome; grey: the facultative-parthenogenesis outcome; black: the obligate-parthenogenesis outcome). Simulation runs are 20,000 generations. Other parameters are as in Figure 6.
(TIF)
