# Supplementary figures and images for: Sexual Conflict over the Maintenance of Sex: Effects of Sexually Antagonistic Coevolution for Reproductive Isolation of Parthenogenesis
Source: PLoS One. 2013 Feb 28;8(2):e58141. doi: 10.1371/journal.pone.0058141 (PMC3585248; doi:10.1371/journal.pone.0058141)

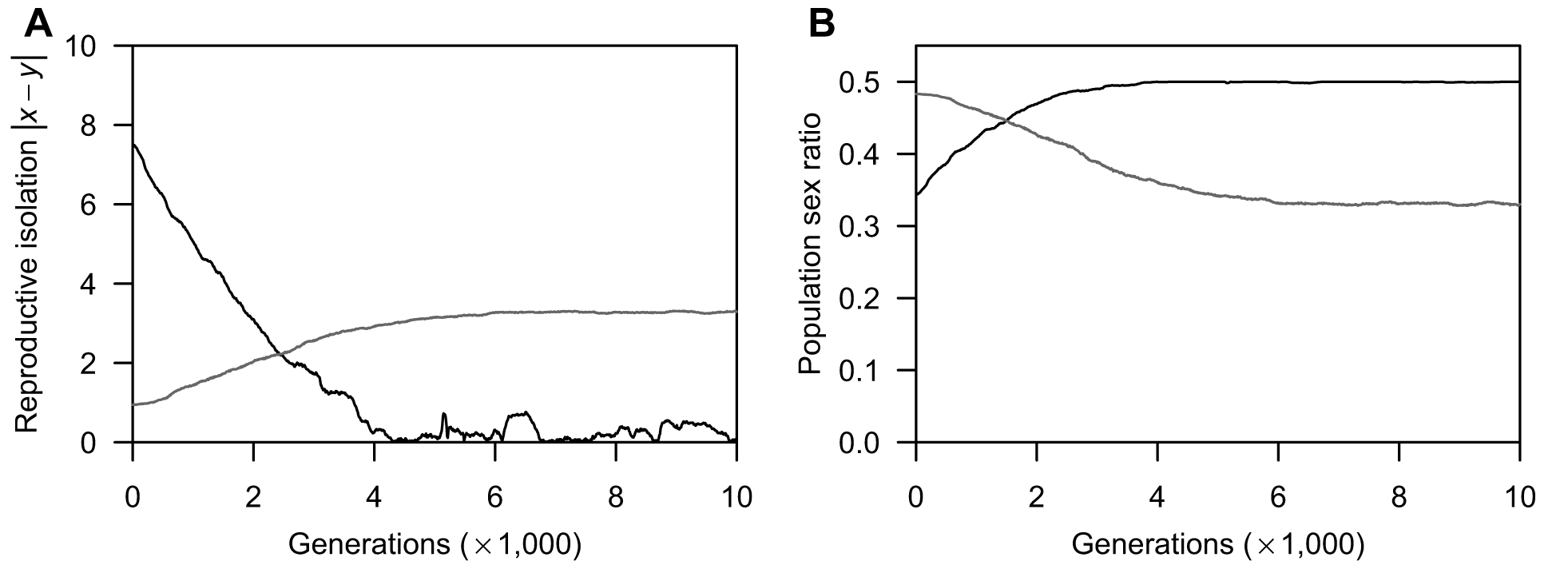

Supplement: Figure S1 — Simulation results of the individual-based model under the condition of facultative parthenogenesis. A: The coevolutionary dynamics of the mean degree of reproductive isolation over 25 simulation runs; B: The demographic dynamics of the mean population sex ratio over 25 simulation runs. The black line indicates the dynamics in a population with higher male PRR (μ = 1.5) that starts at (x, y) = (7.5, 0.0). The grey line indicates the dynamics in a population with lower male PRR (μ = 0.3) that starts at (x, y) = (1.0, 0.0). Other parameters are r = 10.0, h = 0.001, α = 0.01, βm = βf = 0.00. In this analysis, the individual-based model assumes the situation of facultative parthenogenesis: all individuals have only parthenogenetic capacity and the mutation does not occur. (TIF) [file pone.0058141.s001.tif]

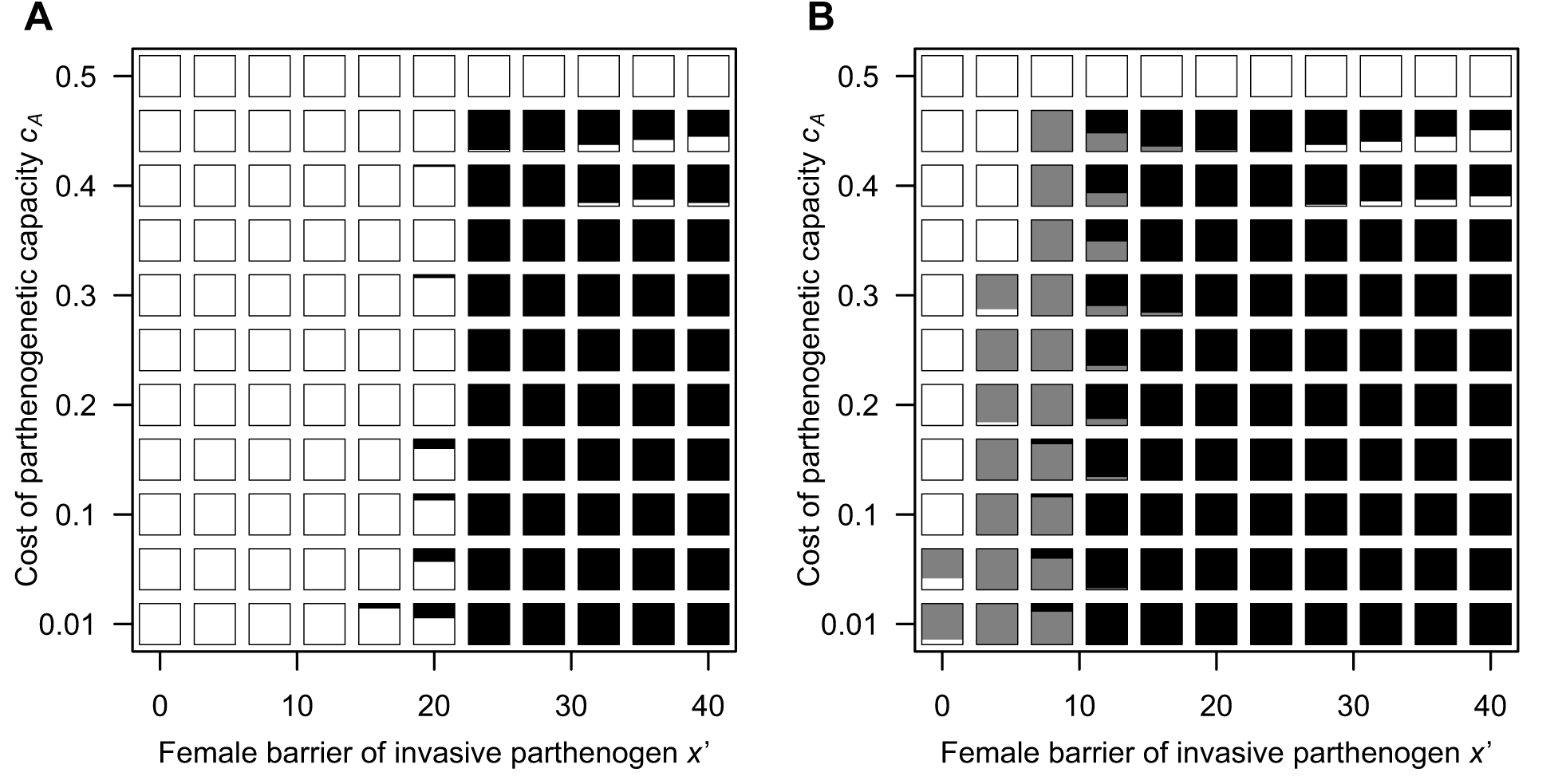

Supplement: Figure S2 — Simulation outcomes of the models with the frequency-dependence under various values of the reproductive barrier of the invasive parthenogens and the cost of parthenogenetic capacity. In this analysis, I have newly incorporated the frequency-dependence on the fertilization probability of females: male mating attempts are dependent on the population sex ratio and the frequency-dependence affects female fertilisation with the form ψ(x, y)M */μF*. As shown here, the frequency-dependence reduces areas of the obligate-sex outcome in comparison with the results of the non-frequency-dependent model (Fig. 6). However, the conclusion of the new model is not quantitatively different with that of the original model: the establishment of obligate parthenogenesis requires that females simultaneously evolve both the capacity for parthenogenesis and complete reproductive isolation from males of parental sexuals. The two panels differ in the value of male PRR (A: μ = 2.00, B: μ = 0.50). Each box indicates the proportion of different outcomes over 25 replicates under its parameter set (white: the obligate-sex outcome; grey: the facultative-parthenogenesis outcome; black: the obligate-parthenogenesis outcome). Simulation runs are 20,000 generations. Other parameters are as in Figure 6. (TIF) [file pone.0058141.s002.tif]
